# Supplementary material for: Molecular Traceability Approach to Assess the Geographical Origin of Commercial Extra Virgin Olive Oil
Source: Foods. 2024 Jul 16;13(14):2240. doi: 10.3390/foods13142240 (PMC11275430; doi:10.3390/foods13142240)
Supplement: Supplementary file 1 [file foods-13-02240-s001.zip › foods-3037861-supplementary.pdf]

Article

Molecular Traceability Approach to Assess the Geographical Origin of Commercial Extra Virgin Olive Oil

Michele Antonio Savoia [1], Isabella Mascio [1], Monica Marilena Miazzi [1], Claudio De Giovanni [1], Fabio Grillo Spina [2], Stefania Carpino [3], Valentina Fanelli [1]*and Cinzia Montemurro [1,4]

[1] Department of Soil, Plant and Food Sciences, University of Bari Aldo Moro, via Amendola 165/A, 70126 Bari, Italy
[2] Department of the Central Inspectorate for the Protection of the Quality and Repression of Fraud of food products ICQRF Via Quintino Sella 42 00187, Roma; Italy
[3] Office PREF IV4 Director, Central Inspectorate for Fraud Repression and Quality Protection of the Agrifood Products and Food, ICQRF, MASAF Via Quintino Sella 42 00187, Roma
[4] Spin off Sinagri s.r.l., University of Bari Aldo Moro, via Amendola 165/A, 70126 Bari, Italy
* Correspondence: valentina.fanelli@uniba.it (V.F)

Supplementary Materials

Table S1. List of 28 EVO oils, labeled and not, analyzed in this study.

Sample Code	Type of sample	Declared origin of the olives
OT1	Not bottled	Tunisia
OT2	Not bottled	Italy
OT3	Not bottled	Tunisia
OT4	Not bottled	Spain
OT5	Not bottled	Tunisia
OT6	Not bottled	Tunisia
OT7	Not bottled	Tunisia
OT8	Not bottled	Italy
OT9	Commercial	EU
OT10	Commercial	EU
OT11	Commercial	Italy, Spain, Greece, and Tunisia
OT12	Commercial	Italy, Spain, and Greece
OT13	Commercial	Italy, Spain, Greece, and Tunisia
OT14	Commercial	Italy, Spain, Greece, and Tunisia
OT15	Commercial	Italy
OT16	Commercial	EU and not-EU
OT17	Not bottled	Tunisia
OT18	Commercial	Italy
OT19	Commercial	Italy
OT20	Commercial	EU
OT21	Commercial	EU and not-EU
OT22	Commercial	Italy
OT23	Commercial	Italy
OT24	Commercial	EU and not-EU
OT25	Commercial	Italy
OT26	Commercial	Italy, Spain, Greece, and Tunisia
OT27	Commercial	Italy, Spain, Greece, and Tunisia
OT28	Commercial	EU and not-EU

The declared origin of olives used for oil production is also indicated.

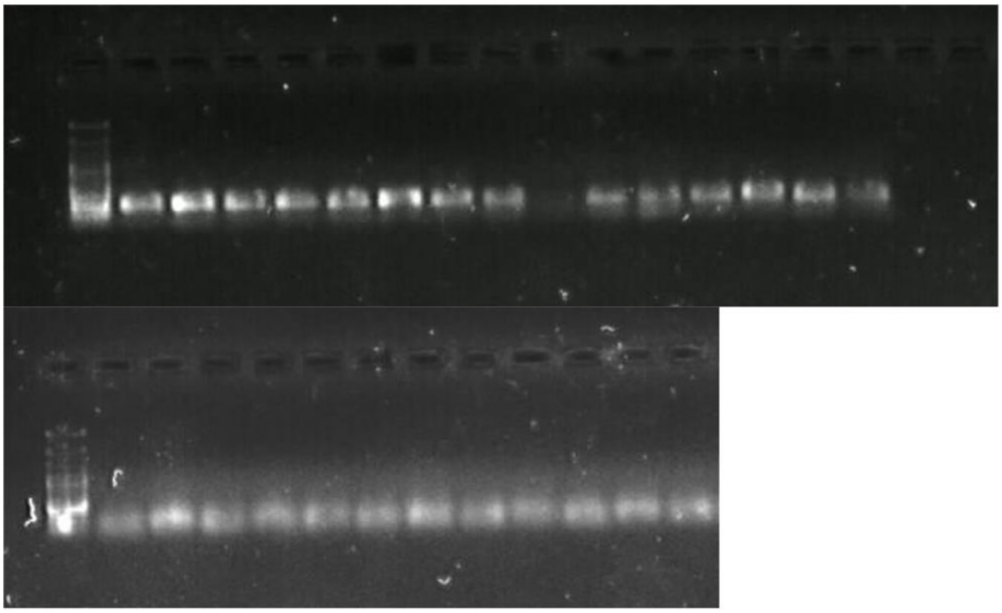

Figure S1. Agarose gel electrophoresis of PCR products obtained using oil samples from OT1 to OT17, first row, and from OT18 to OT28, second row (last sample is the positive control) amplified with primers corresponding to DCA18 marker (the used ladder is the GeneRuler 100 bp).
